# Not one-size-fits-all: diabetes in south Asia

**DOI:** 10.1016/j.lansea.2025.100694

**Published:** 2025-11-11

**Authors:** The Lancet Regional Health – Southeast Asia

November 14th marks World Diabetes Day. As of 2025, an estimated 589 million adults worldwide are living with diabetes. The Internation Diabetes Federation (IDF) reports that 107 million adults in our region are affected by diabetes. India alone accounts for 89 million cases, making it the country with the second-largest diabetes burden globally. Over the past three decades, the region has experienced a sharp increase in diabetes prevalence, disability, and mortality, and projections up to 2050 indicate that this upward trend is likely to persist. In south Asia it is estimated that almost half of the adults living with diabetes are undiagnosed. In India, the largest conducted epidemiological study on diabetes demonstrated that fewer than one in four people on regular treatment had achieved glycaemic control (defined in the study as HbA1c <7%). This is mirrored in other countries from the region, underscoring an unmet need. Recognising this significant gap—by examining recent advances in diabetes classification, the limitations of imported treatment guidelines, and the importance of holistic, culturally relevant care—we underscore the necessity of tailored approaches that can improve outcomes for the millions living with diabetes in our region.

For instance, research from India has highlighted that type 2 diabetes in south Asians represents a distinct phenotype—the “south Asian phenotype”, which is characterised by earlier onset of illness, occurring at lower BMI, and associated with central obesity, higher visceral adiposity, lower lean body mass, and accelerated β-cell dysfunction. This dual burden of insulin resistance and impaired insulin secretion leads to faster progression from prediabetes to overt diabetic state in south Asian populations.

Recent advances in diabetes classification reinforce this heterogeneity as a defining feature of diabetes in south Asia. Through cluster analysis, the ANDIS project from Sweden identified five distinct subtypes of adult-onset diabetes with distinct clinical presentations, trajectories, and responses to treatment: severe autoimmune diabetes, severe insulin-deficient diabetes (SIDD), severe insulin-resistant diabetes, mild obesity-related diabetes, and mild age-related diabetes. When this was applied in Indian cohorts, the severely insulin-resistant group exhibited additional characteristics of poor β-cell function and higher levels of obesity—features not seen in the Scandinavian group. In south Asia, the SIDD subtype is more prevalent, especially among young individuals with low BMI. In an Indian cohort, insulin-deficient subtypes comprised up to 75% of type 2 diabetes.

In another shift, a landmark consensus in January 2025 recognised a distinct phenotype of diabetes, long neglected since its first description in 1955. Termed type 5 diabetes, this is seen in lean individuals with a life course history of undernutrition and associated with lower C-peptide concentration, severely impaired insulin secretion and preserved insulin sensitivity. This decision was formally endorsed by the IDF World Diabetes Congress in April 2025. Type 5 diabetes is thought to result from malnutrition-related impaired pancreatic development and is largely found in low-income and middle-income countries. This is significant as existing treatment protocols for more common forms of diabetes (type 1 and type 2) are inadequate for this phenotype, which needs a tailored approach. Thought to affect an estimated 20–25 million people worldwide, we lack clarity on prevalence and impact in south Asia. To improve our understanding of this phenotype, the IDF has established a Working Group with a primary aim being to develop formal diagnostic criteria for the condition.

While these advances illuminate the unique nature of our regional diabetes burden, they also reveal a significant evidence gap; in many South Asian countries, reliance on frameworks from the IDF or WHO persists, despite their limited relevance to the needs and diversity of the region. Existing national guidelines incorporate local epidemiological data, yet rely heavily on evidence from other regions for therapeutic recommendations—even as fundamental criteria such as HbA1c thresholds are debated in Indian populations. Further, commonly used parameters such as BMI are increasingly recognised as inadequate for assessing diabetes risk in south Asian populations. Rural, tribal, and other marginalised groups remain under-represented in current datasets, further contributing to the evidence gap.

Without robust, population-specific data on diabetes phenotypes, diagnosis and treatment protocols are likely to remain unsuited to the specific needs of the region, resulting in subpar outcomes. Translational and population-based research in south Asia is urgently needed to capture the full spectrum of diabetes, including genetic diversity, environmental factors, and socioeconomic determinants. Local clinical trials can inform the creation of adapted guidelines, improve screening strategies, and facilitate the development of targeted therapies that address the heterogeneity of diabetes in the region.

In addition to generating regional evidence, there is an urgent need for heightened clinical awareness of diabetes subtypes, which calls for a more precise approach to diagnosis and management. Functional classification based on C-peptide testing and other routine markers allows clinicians to distinguish between insulin-deficient and insulin-resistant states, leading to clinically actionable insights. However, these efforts would be ineffective without culturally relevant advice and strategies to improve adherence to medication and engagement with health services. A nuanced approach—incorporating caregiver involvement, targeted education, and regular screening for complications—can empower individuals and significantly improve long-term outcomes for people living with diabetes.

Advances in understanding highlight both challenges and opportunities. With the scale and distinct character of the diabetes burden, this challenge is not something to be ignored. We at *The Lancet Regional Health—Southeast Asia* are keen to publish research that has the potential to impact the 107 million people living with diabetes in the region. With robust evidence that reflects the genetic, metabolic, and cultural diversity, we can ultimately aim to tailor prevention and treatment strategies—transforming a regional liability into a lesson for the rest of the world.

